# A novel focal adhesion related gene signature for prognostic prediction in hepatocellular carcinoma

**DOI:** 10.18632/aging.202871

**Published:** 2021-04-13

**Authors:** Zhuo Lin, Dan Miao, Qian Xu, Xiaodong Wang, Fujun Yu

**Affiliations:** 1 Department of Hepatology, The First Affiliated Hospital of Wenzhou Medical University, Wenzhou, Zhejiang, China; 2 Zhejiang Provincial Key Laboratory for Accurate Diagnosis and Treatment of Chronic Liver Diseases, Wenzhou, Zhejiang, China; 3 Department of Gastroenterology, the First Affiliated Hospital of Wenzhou Medical University, Wenzhou, China

**Keywords:** hepatocellular carcinoma, focal adhesion, gene signature, overall survival, immune status

## Abstract

Hepatocellular carcinoma (HCC) is a highly heterogeneous disease. Reduced expression of focal adhesion is considered as an important prerequisite for tumor cell invasion and metastasis. However, the prognostic value of focal adhesion related genes in HCC remains to be further determined. In this study, RNA expression profiles were downloaded from public databases. A five focal adhesion related gene signature model was established by the least absolute shrinkage and selection operator Cox regression analysis, which categorized patients into high- and low-risk groups. Multivariate Cox regression analysis showed that the risk score was an independent predictor for overall survival. Single-sample gene set enrichment analysis revealed that immune status was different between the two risk groups, and tumor-related pathways were enriched in high-risk group. The risk score was significantly associated with tumor grade, tumor stage, immune scores, and immune infiltrate types. Pearson correlation showed that the expression level of prognostic genes was associated with anti-tumor drug sensitivity. Besides, the mRNA and protein expression of prognostic genes was significantly different between HCC tissues and adjacent non-tumorous tissues in our separate cohort. Taken together, a novel focal adhesion related gene signature can be used for prognostic prediction in HCC, which may be a therapeutic alternative.

## INTRODUCTION

Liver cancer is the third global leading cause of cancer-related death globally with a high mortality rate [1]. As the main histologic type of liver cancer, hepatocellular carcinoma (HCC) accounts for approximately 90% of all liver cancers and causing approximately 750,000 deaths annually [2, 3]. In most developing countries, hepatitis B virus (HBV) and hepatitis C virus (HCV) infections are the main risk factors of HCC, while in most developed countries, alcohol abuse and metabolic syndrome-related diabetes and obesity are the main underlying disorders [4]. Surgical resection, liver transplantation, and radiofrequency ablation remain the mainstay of treatment for HCC [5, 6]. Although an increasing number of diagnostic and therapeutic strategies have been developed, the prognosis of HCC patients remains poor due to the elusive molecular mechanisms. It is therefore necessary to gain further insights into the molecular mechanisms underlying HCC progression and seek novel prognostic biomarkers and therapeutic strategies for this fatal disease.

Focal adhesion, as the main connection between cells and the extracellular matrix (ECM), helps maintain the tension of cells during movement and signal transmission of cell survival [7, 8]. Of various microenvironmental factors affecting cancer cell resistance, cell adhesion to the ECM has recently been identified as a key determinant [9]. Reduced expression of focal adhesion promotes the process of epithelial-mesenchymal transition and is considered as an important prerequisite for tumor cell invasion and metastasis [10–14]. Focal adhesion molecules including integrins, focal adhesion kinases, and growth factor receptors have been shown to be associated with cancer progression [15–17]. Besides, more and more research has demonstrated that focal adhesion related genes such as ITGA [18, 19], NEK2 [20–22], and cPLA2α [23, 24] all play a promoting role in HCC. However, whether the focal adhesion related genes are associated with the prognosis of HCC patients remains unclear. Given the most upstream localization, receptors within focal adhesions were supposed to be an ideal starting point for therapeutic intervention [9], suggesting that focal adhesion may prove to be the prognostic indicator and a novel therapeutic target in HCC patients.

In this study, we developed a prognostic signature for HCC patients based on focal adhesion related genes. We also investigated the correlation of the prognostic model risk score with the clinicopathological characteristics. In addition, we analyzed the role of prognostic model risk scores in immune infiltrate types, tumor stemness features, and prognostic gene expression in drug chemoresistance. Also, the stability and reliability of the model were demonstrated in an independent and external validation cohort, and the mRNA and protein expressions of the prognostic genes between HCC and adjacent non-tumorous tissues were verified by our laboratory experiments. It is our hope that the findings of the present study could provide a novel prognostic model and therapeutic targets for HCC.

## RESULTS

**Figure 1:**
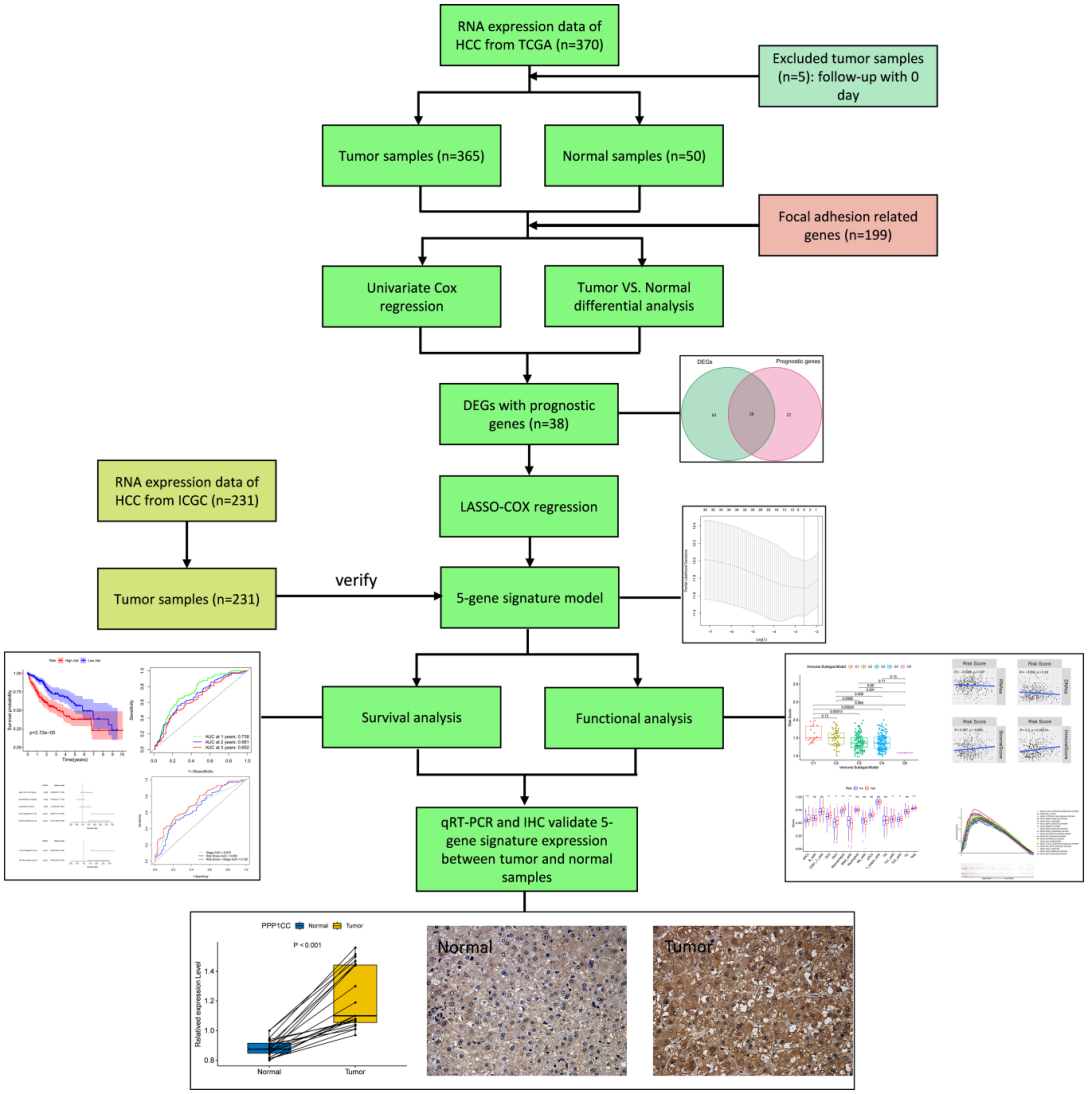
**Flow chart of data collection, analysis and experiment.**

The flow chart of this study is demonstrated in Figure 1. Altogether 365 HCC patients from The Cancer Genome Atlas hepatocellular carcinoma cohort (TCGA-LIHC) and 231 HCC patients from the International Cancer Genome Consortium hepatocellular carcinoma cohort (ICGC-LIRI-JP) were eventually recruited for analysis. The clinicopathological characteristics of these patients are detailed in Table 1.

**Table 1:** Clinical characteristics of the HCC patients used in this study.

	**TCGA-LIHC cohort**	**ICGC-LIRP-JI cohort**
**No. of patients**	365	231
**Age (median, range)**	57 (16–90)	67 (31–89)
**Gender**		
Female	119 (32.6%)	61 (26.4%)
Male	246 (67.4%)	170 (73.6%)
**Grade**		
Grade 1	55 (15.1%)	NA
Grade 2	175 (47.9%)	NA
Grade 3	118 (32.3%)	NA
Grade 4	12 (3.3%)	NA
Unknown	5 (1.4%)	NA
**Stage**		
I	170 (46.6%)	36 (15.6%)
II	84 (23.0%)	105 (45.5%)
III	83 (22.7%)	71 (30.7%)
IV	4 (1.1%)	19 (8.2%)
Unknown	24 (6.6%)	0 (0%)
**Survival status**		
Alive	235 (64.4%)	189 (81.8%)
Deceased	130 (35.6%)	42 (18.2%)

### Identification of DEGs in the TCGA cohort

**Figure 2:**
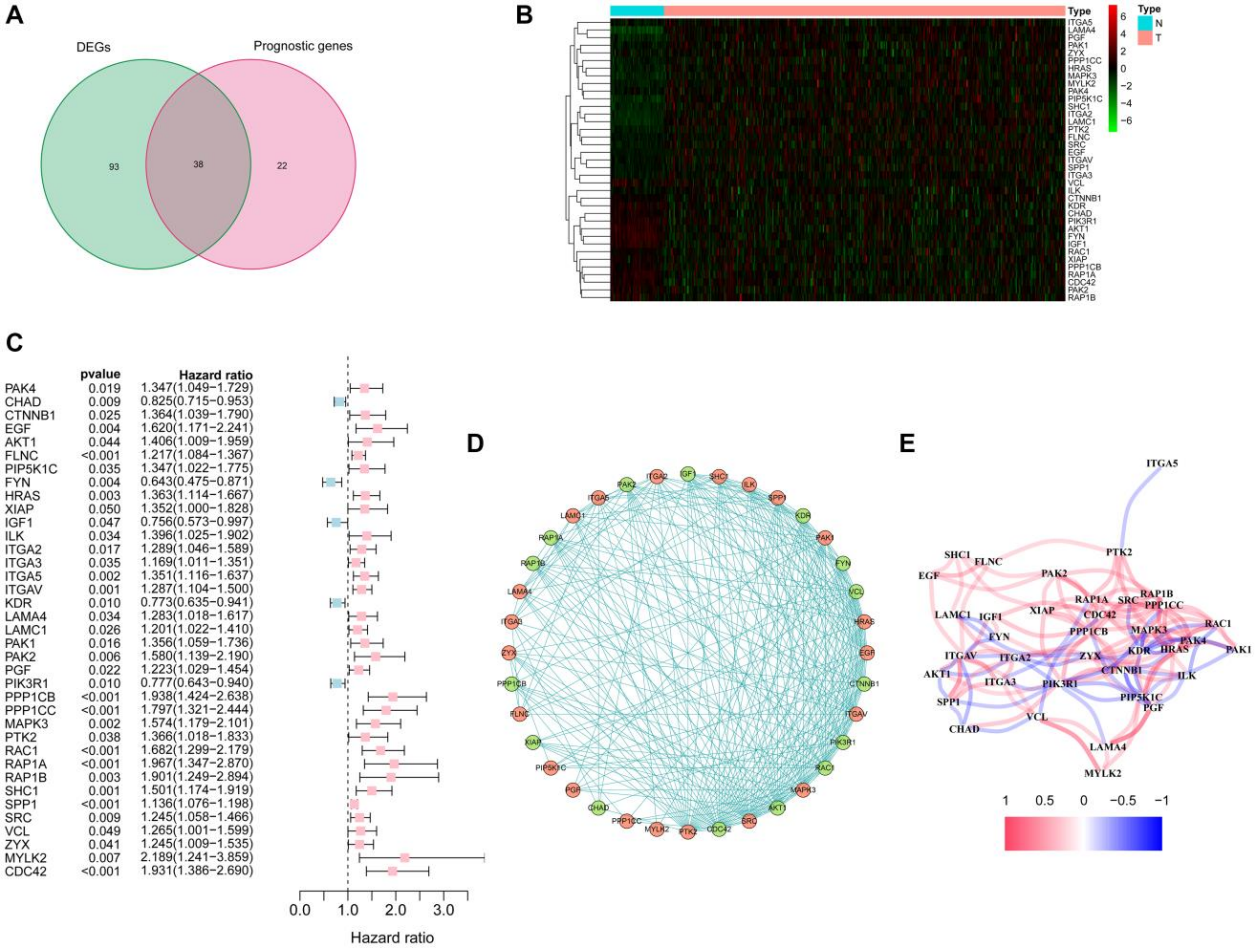
**Identification of candidate focal adhesion related genes in the TCGA cohort.** (**A**) Venn diagram to identify DEGs between HCC and adjacent normal tissues. (**B**) Expression of the 37 overlapping genes between HCC and adjacent normal tissues. (**C**) Forest plots showing the results of the univariate Cox regression analysis between the expression of 37 overlapping genes and overall survival. (**D**) The protein-protein interaction network indicated the interactions between the candidate genes. (**E**) The correlation network of candidate genes.

More than 50% of focal adhesion related genes (131/199, 65.8%) were identified as differentially expressed genes (DEGs) between the tumor and adjacent non-tumorous tissues using the "limma" R package, and Univariate Cox regression analysis showed that 38 of the 131 DEGs were correlated with overall survival (OS) (Figure 2A). As the expression level of ACTN3 was 0 in more than 80 samples, it was excluded from this analysis. Finally, 37 focal adhesion related DEGs were preserved (Figure 2B–2C). The protein-protein interaction network and correlation network between these DEGs are shown in Figure 2D–2E.

### Construction of a prognosis model in the TCGA cohort

**Figure 3:**
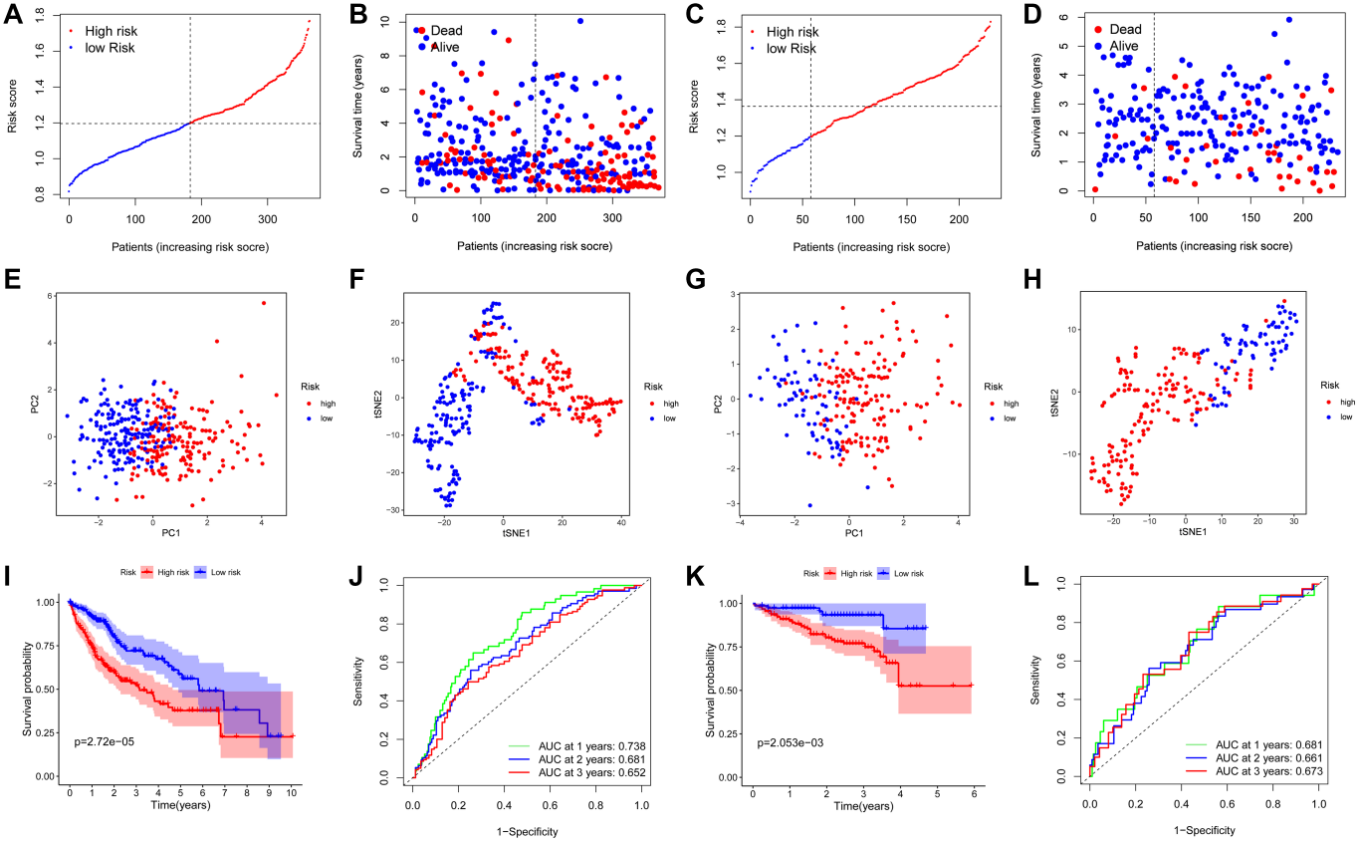
**Prognostic analysis of the 5-gene signature model in the TCGA cohort and ICGC cohort.** TCGA cohort (**A**, **B**, **E**, **F**, **I**, **J**), ICGC cohort (**C**, **D**, **G**, **H**, **K**, **L**). (**A**, **C**) The distribution and median value of the risk scores. (**B**, **D**) Distributions of the overall survival (OS) status. (**E**, **G**) PCA plot. (**F**, **H**) t-SNE analysis. (**I**, **K**) Kaplan-Meier curves for OS of patients in the high-risk group and low-risk group. (**J**, **L**) Time-dependent ROC curves for OS.

The least absolute shrinkage and selection operator (LASSO) Cox regression analysis was performed to construct a prognosis model based on the expression profiles of the above-mentioned 37 DEGs. Using the optimal value of λ, a 5-gene signature was identified (Supplementary Figure 1). The risk score was calculated by using the following equation: Score = ˗0.031 * expression level of *FYN* + 0.170 * expression level of *PPP1CB* + 0.003 * expression level of *PPP1CC* + 0.084 * expression level of *RAC1* + 0.045 * expression level of *SPP1.* The patients were categorized into a high-risk group and a low-risk group based on the median cut-off value (Figure 3A). The tumor grades were higher and the TNM stages were more advanced in the TCGA cohort of high-risk group as compared with those in low-risk group (Table 2). PCA and t-SNE analyses showed that patients in these different risk groups were distributed in two directions (Figure 3E–3F). As shown in Figure 3B, death usually occurred earlier in high-risk group than that in low-risk group (*P* = 0.007). Conformably, the Kaplan-Meier curve indicated that the prognosis of high-risk patients was significantly poorer than that of low-risk patients (*P* < 0.001) (Figure 3I). The predictive performance of the risk score for OS was evaluated by time-dependent ROC curves. The area under the curve (AUC) of the prognosis model at 1, 2 and 3 years was 0.738, 0.681 and 0.652, respectively, indicating a good predictive performance (Figure 3J). Survival analysis using the optimal cut-off expression value of each prognostic gene showed that high expressions of *PPP1CB*, *PPP1CC*, *RAC1* and *SPP1* were correlated with poor prognoses, whereas *FYN* showed an opposite correlation (*P* < 0.05) (Supplementary Figure 2A–2E). The different expression of each prognostic gene between HCC and adjacent non-tumorous tissues in the TCGA cohort is shown in Supplementary Figure 3.

**Table 2:** Baseline characteristics of the patients in different risk groups.

**Characteristics**	**TCGA-LIHC cohort**			**ICGC-LIRP-JI cohort**		
	**High risk**	**Low risk**	***P* value**	**High risk**	**Low risk**	***P* value**
**Age**						
< 60 year	74 (20.3%)	91 (24.9%)	0.082	28 (12.1%)	16 (6.9%)	0.540
≥ 60 year	108 (29.6%)	92 (25.2%)		128 (55.4%)	59 (25.5%)	
**Gender**						
Female	61 (16.7%)	58 (15.9%)	0.710	40 (17.3%)	21 (9.1%)	0.703
Male	121 (33.2%)	125 (34.2%)		116 (50.2%)	54 (23.4%)	
**Grade**						
G1+G2	104 (28.5%)	126 (34.5%)	0.016	–	–	
G3+G4	76 (20.8%)	54 (14.8%)		–	–	
unknown	2 (0.5%)	3 (0.8%)		–	–	
**Stage**						
I + II	116 (31.8%)	138 (37.8%)	0.023	86 (37.2%)	55 (23.8%)	0.008
III + IV	52 (14.2%)	35 (9.6%)		70 (30.3%)	20 (8.7%)	
unknown	14 (3.8%)	10 (2.7%)		0 (0%)	0 (0%)	

### Confirmation of the 5-gene signature in the ICGC cohort

The predictive stability of this prognosis model was further verified by using HCC samples from the ICGC database. Consistent with the above results, the HCC patients categorized into a high-risk group and a low-risk group based on the median value from the TCGA cohort (Figure 3C). The TNM stage was more advanced in in the ICGC cohort of high-risk group (Table 2). PCA and t-SNE analyses demonstrated that patients in the two subgroups were distributed discretely (Figure 3G–3H). Patients in high-risk group died earlier (Figure 3D) and had shorter survival durations (Figure 3K) than patients in low-risk group (both *P* < 0.05). Additionally, the AUC of the 5-gene signature at 1, 2 and 3 years was 0.681, 0.661 and 0.673, respectively (Figure 3L). The result of survival analysis of each prognostic gene with high or low expression is shown in Supplementary Figure 2F–2J.

### The independent prognostic value of the 5-gene signature

**Figure 4:**
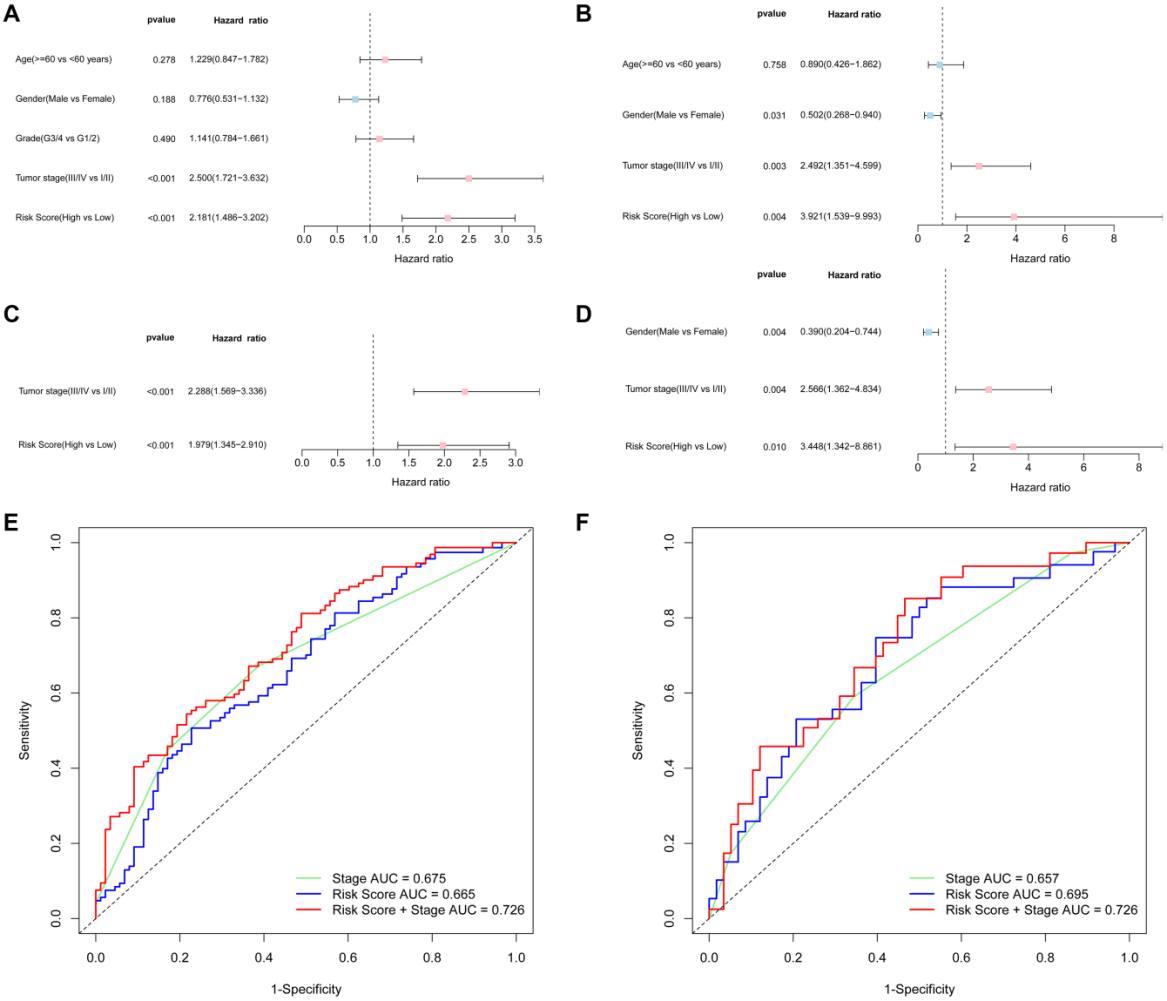
**Results of the univariate and multivariate Cox regression analyses regarding overall survival (OS) and AUC of the risk score, tumor stage, and the risk score combined with tumor stage at 3-year OS.** TCGA cohort (**A**, **C**, **E**), ICGC cohort (**B**, **D**, **F**). (**A**, **B**) Univariate Cox regression analyses to screen OS-related factors. (**C, D**) Multivariate Cox regression analyses to screen OS-related factors. (**E**, **F**) AUC of the risk score, tumor stage, and the risk score combined with tumor stage at 3-year OS.

Univariate and multivariate Cox regression analyses were performed to confirm whether the risk score was independent of traditional clinicopathological characteristics for OS. Univariate Cox regression showed that the tumor stage (TCGA cohort: HR = 2.500, 95% CI = 1.721–3.363, *P* < 0.001; ICGC cohort: HR = 2.492, 95% CI = 1.351–4.599, *P* = 0.003) and the risk score (TCGA cohort: HR = 2.181, 95% CI = 1.486–3.202, *P* < 0.001; ICGC cohort: HR = 3.921, 95% CI = 1.539–9.993, *P* = 0.004) were independent factors for predicting the OS prognosis (Figure 4A–4B). With other confounding factors adjusted, the results were consistent with the above (Figure 4C–4D). Furthermore, the AUC of the risk score combined with the tumor stage at 3-year OS (TCGA set: AUC = 0.726; ICGC set: AUC = 0.726) demonstrated that the combined model worked the best for predicting OS (Figure 4E–4F).

### Relationship between the clinical characteristics and risk score

**Figure 5:**
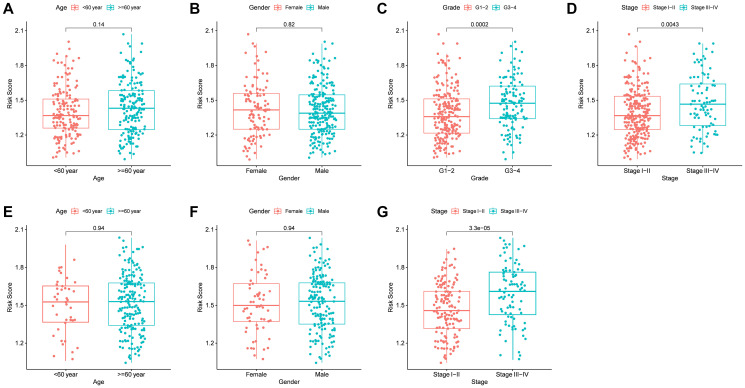
**The risk score in different groups stratified by clinical characteristics.** TCGA cohort (**A**–**D**), ICGC cohort (**E**–**F**). (**A**, **E**) Age. (**B**, **F**) Gender. (**C**) Tumor grade. (**D**, **G**) Tumor stage.

The correlation between the risk score and various clinical characteristics in HCC patients based on TCGA and ICGC data is indicated in Figure 5A–5G, respectively. Higher risk score was observed in patients with the higher tumor grades (*P* < 0.001) and more advanced tumor stage (*P* = 0.004) in the TCGA cohort. However, the risk score had no significant correlation with age and gender. The same analysis in the ICGC cohort showed the similar results (There were no data about the grade of LICH in the ICGC cohort). Further analysis of the correlation of the expression level of the prognostic genes with age, gender, tumor stage and tumor grade of HCC patients showed that the expression of *FYN*, *PPP1CC*, *RAC1*, and *SPP1* was significantly correlated with the tumor grade (*P* < 0.05). In addition, the expression of *PPP1BC*, *PPP1CC* and *RAC1* was correlated with the tumor stage (*P* < 0.05) (Supplementary Figure 4C–4D); the expression of *PPP1CC* and *SPP1* was correlated with age (*P* < 0.05) (Supplementary Figure 4A); the expression of *PPP1CB* was correlated with gender (*P* < 0.05) (Supplementary Figure 4B).

### Analysis of the immune status and tumor microenvironment

**Figure 6:**
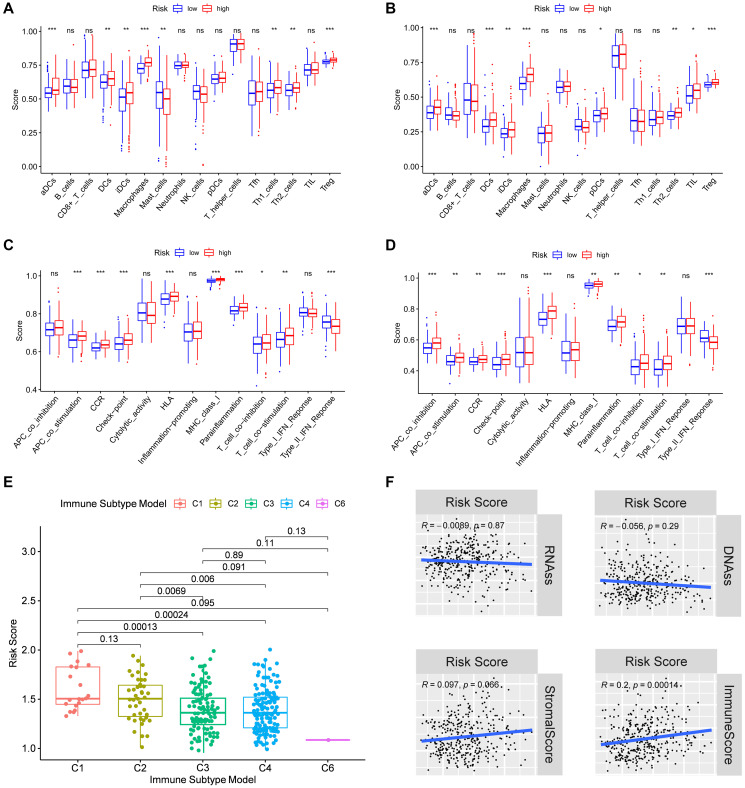
**Immune status between different risk groups and the association of risk score with the tumor microenvironment.** TCGA cohort (**A**, **C**), ICGC cohort (**B**, **D**). (**A**, **B**) The scores of 16 immune cells. (**C**, **D**) The boxplots showing the 13 immune-related functions. (**E**) Comparison of the risk scores between different immune infiltrate subtypes. (**F**) The relationship of risk score with RNAss, DNAss, Stromal Score and Immune Score. *P* values are showed as: ns, not significant; ^*^P < 0.05; ^**^P < 0.01; ^***^P < 0.001.

To further explore the immune status in different risk groups, the enrichment scores of diverse immune cell subpopulations and related functions were quantitated by single-sample gene set enrichment analysis (ssGSEA). The ratios of aDCs, DCs, iDCs, macrophages, Th2 cells, and Treg were high in patients of high-risk group, and vise versa for the score of Mast cells (*P* < 0.05) (Figure 6A–6B). Similarly, the score of immune-related functions such as APC co-inhibition, APC co-stimulation, Check point, HLA, MHC class I, Parainflammation, T cell co-inhibition, and T cell co-stimulation was significantly higher in high-risk group, and vise versa for the score of Type II IFN (*P* < 0.05) (Figure 6C–6D). All these results suggest that the immune status and tumor microenvironment might contribute to the prognosis of HCC patients with high expressions of focal adhesion related genes.

Subsequently, we examined the risk score distribution in different immune subtypes in HCC patients. Of the six types of immune infiltrates identified in human tumors [C1 (wound healing), C2 (INF-γ dominant), C3 (inflammatory), C4 (lymphocyte depleted), C5 (immunologically quiet), and C6 (TGF-β dominant)] [25], and no patient sample belonged to the C5 immune subtype in HCC. The value of the risk score from C1 to C6 subtype decreased gradually. As shown in Figure 6E, the high-risk score was significantly correlated with C1 and C2, and the low-risk score was correlated with C3, C4 and C6 (*P* < 0.05). Furthermore, the high levels of *PPP1CB, PPP1CC, RAC1, and SPP1* were positively correlated with C1, suggesting their promoting roles in HCC. On the contrary, the high level of *FYN* was negatively correlated with the C1 (*P* < 0.05) (Supplementary Figure 5), suggesting its tumor-suppressive role in HCC.

To explore whether the risk score was related to tumor stem cells and the immune microenvironment, the result of correlation analysis was showed that the risk score was positively correlated with immune scores, suggesting an association of the prognostic model with immune composition (*P* < 0.001). On the contrary, the risk score had no significant correlation with stemness score based on mRNA expression (RNAss), DNA methylation based stemness score (DNAss), and stromal score (Figure 6F). Moreover, the correlations of tumor stem cells and the immune microenvironment with prognostic gene expression were analyzed. It was found that *FYN, PPP1CB,* and *PPP1CC* were correlated with RNAss, while *FYN, PPP1CC,* and *SPP1* were correlated with the stromal score (*P* < 0.05), where F*YN* exhibited strongest correlation (R = 0.55) (Supplementary Figure 6). Furthermore, *FYN, RAC1,* and *SPP1* were found to be positively correlated with the immune score which measures the presence of infiltrating immune cells (*P* < 0.05) (Supplementary Figure 6).

### Analysis of biological functions and pathways

**Figure 7:**
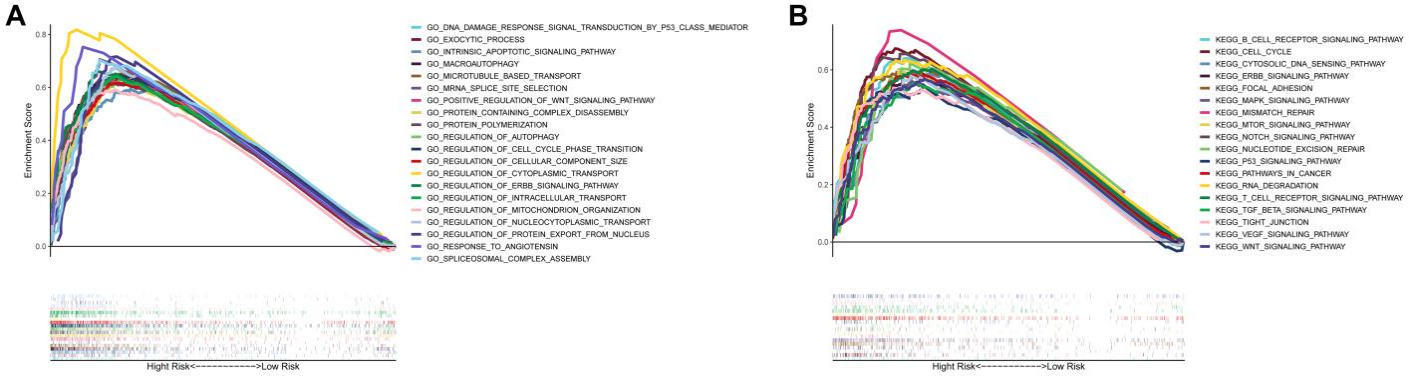
**Gene set enrichment analysis of biological functions and pathways.** (**A**) GO, Gene Ontology. (**B**) KEGG, Kyoto Encyclopedia of Genes and Genomes.

Using gene set enrichment analysis (GSEA), gene ontology (GO) enrichment and Kyoto encyclopedia of genes and genome (KEGG) pathways were analyzed in high- and low-risk groups. It was found that 20 main GO functions were enriched in high-risk group with a false discovery rate <0.05. The enriched GO functions showed that the risk score was significantly associated with the activations of regulation of autophagic regulation, cell cycle phase transition, intrinsic apoptotic signaling pathway, and positive regulation of Wnt signaling pathway (Figure 7A). As shown by KEGG pathway analysis, the pathway enrichment was correlated with the cancer procession including cell cycle, MAPK signaling pathway, mismatch repair, mTOR signaling pathway, notch signaling pathway, P53 signaling pathway, pathways in cancer, VEGF signaling pathway, and Wnt signaling pathway. Of note, the focal adhesion pathway was also enriched in high-risk group (Figure 7B). These results indicate that the prognostic model had an extensive influence of on the global transcriptome of HCC tissues.

### Chemotherapy sensitivity analysis

**Figure 8:**
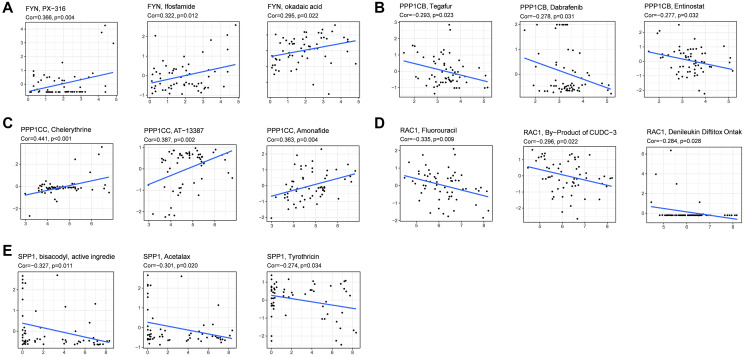
**Scatter plots of the association between prognostic gene expression and drug sensitivity.** (**A**) FYN. (**B**) PPP1CB. (**C**) PPP1CC. (**D**) RAC1. (**E**) SPP1.

Then we investigated the association between focal adhesion related genes and chemotherapy sensitivity in the NCI-60 database. The results showed that all the prognostic genes were to some extent correlated with chemotherapy drug sensitivity (Figure 8, Supplementary Table 4). We also found that the increased expression of *PPP1CB, RAC1,* and *SPP1* was correlated with increased drug resistance of cancer cells to several chemotherapy drugs, such as Tegafur, Dabrafenib, Entinostat, Fluorouracil, By-Product of CUDC-3, Denileukin Diftitox Ontak, Bisacofyl (active ingredient), Acetalax and Tyrothricin (cor > 0.27 and *P* < 0.05). Furthermore, increased *PPP1CC* and *FYN* expression was correlated with increased drug sensitivity to cancer cells, such as PX−316, Ifosfamide, okadaic acid, Chelerythrine, AT-13387, and Amonafide (cor > 0.29 and *P* < 0.05). These results demonstrate that the model could serve as a potential predictor for chemotherapy sensitivity.

### Verification of the expression of prognostic genes between HCC and adjacent non-tumorous tissues

**Figure 9:**
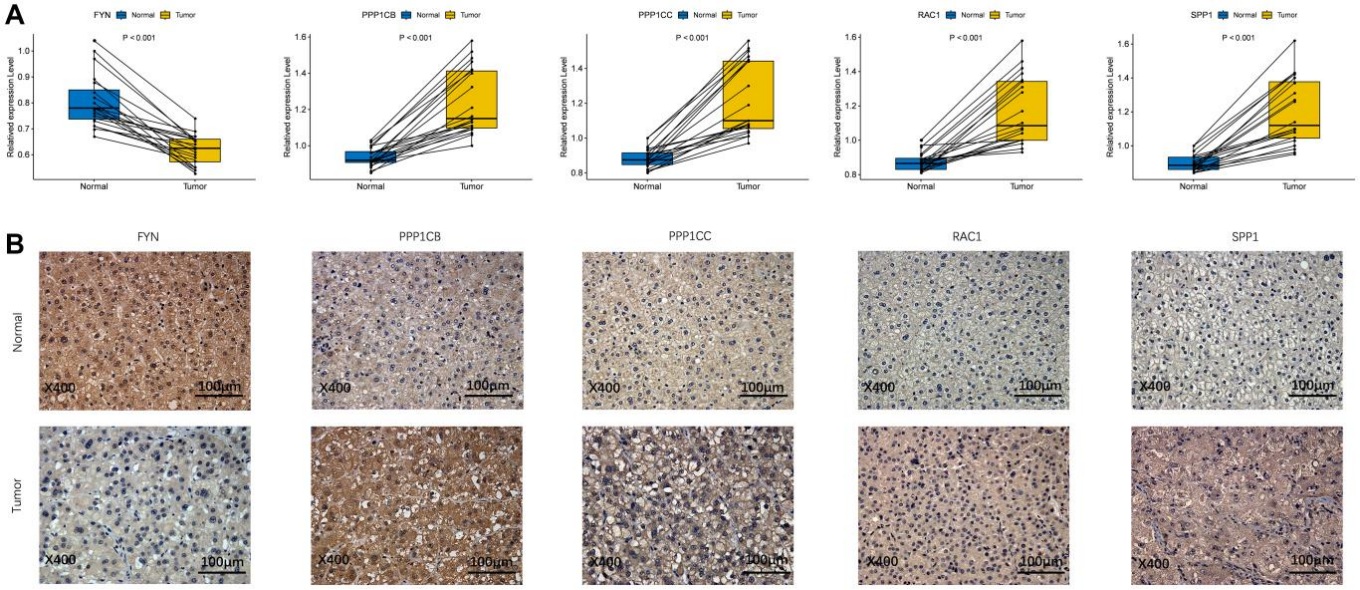
**The expression of prognostic genes between HCC and adjacent non-tumorous tissues.** (**A**) mRNA expression analysis by qRT-RCR. (**B**) Protein expression analysis by IHC.

To validate the different expression of the five prognostic genes *(FYN, PPP1CB, PPP1CC, RAC1,* and *SPP1*) between HCC and adjacent non-tumorous tissues, real-time quantitative-polymerase chain reaction (qRT-PCR) and immunohistochemistry (IHC) were performed to analyze the mRNA and protein expression, respectively. As shown in Figure 9A, *PPP1CB, PPP1CC, RAC1,* and *SPP1* were highly expressed in HCC tissues vs. adjacent non-tumorous tissues, while *FYN* was at a low level in HCC tissues. Immunohistochemical staining showed the same results (Figure 9B). The validation results were consistent with the RNA sequencing results of the five prognostic genes in the TCGA cohort (Supplementary Figure 3).

## DISCUSSION

HCC is one of the most frequent cancer type worldwide [26]. Although multiple molecular mechanisms have been found to be involved in HCC progression [27], the underlying mechanism remains unclear. Our study provided a systematical analysis of the different expression of 199 focal adhesion related genes between HCC and adjacent non-tumorous tissues and their relationships with OS. Then, a 5-gene signature model was constructed by the LASSO algorithm. Both the internal and external validation cohorts demonstrated that the model worked stably and exhibited consistent predictive performance. More importantly, it could be used as an independent risk factor for predicting the prognosis of HCC patients.

Among the prognostic genes, *PPP1CB, PPP1CC, RAC1,* and *SPP1* were found as risk factors, and *FYN* was found as a protective factor. *FYN* is a member of the Src family kinases, which is involved in the focal adhesion signaling pathway. It was reported to be related to numerous solid tumors, and increased expression of FYN was found to play a promoting role both in cancer occurrence and progression, as well as be a mechanism of resistance to anticancer agents [28] such as breast cancer [29], and pancreatic cancer [30]. *PPP1CB* is a regulator of endothelial cell migration and plays a critical role in the angiogenic process. Lacobazzi et al. reported that *PPP1CB* inhibition could inhibit endothelial cell migration through focal adhesion turnover via the actin polymerization pathway [31]. Besides, recent studies have demonstrated that *PPP1CC* encodes protein phosphatase 1c, which may promote cancer cell proliferation through activating mutations in p53 [32]. *RAC1* is identified as a master regulator of cell migration and anchorage-independent growth [33]. Earlier studies reported that SPP1 could promote cancer progression through modulating the expression of VEGF and regulating ECM protein [34, 35]. Interestingly, all the five genes that we analyzed above were all enriched in the focal adhesion signaling pathway which is the molecular bridge mediating two-way crosstalk between ECM and cytoskeleton [36]. But other studies argued that focal adhesion signaling pathway promoted tumor progression and metastasis [37, 38]. Therefore, we subjected the 5-gene signature model to predict the prognosis for HCC, anticipating that it could become a target for cancer drug therapy. We demonstrated that the 5-gene signature model could add prognostic value to the clinicopathological prognostic characteristics.

GSEA enrichment analysis suggested the existence of potential biological processes and pathways in high-risk group. Consistently, our study identified the enrichment in the high-risk-score group including the MAPK, notch, P53, VEGF and Wnt signaling pathways. All these pathways are classical signaling pathways that have been identified to be involved in cancer process [39–41]. The Wnt signaling pathway is severely dysregulated in solid and non-solid malignant tumors, acting as part of the proliferation pathway that characterizes cancer cells [42]. Pérez-Plasencia et al. reported that the Wnt signaling pathway, as a common pathway in many cancer types, was a main attractive target in cancer therapy [43], including HCC [35]. Dysregulation of the MAPK cascade is linked to some key signaling components and phosphorylation events which can activate the process of tumorigenesis [44]. At least three different MAPK signal transduction pathways participate in the modulation and transduction of extracellular signals into the nucleus to induce response genes in such mammalian cells as ERK1/2, JNK1/2/3, and p38 [44, 45], and all of them have proved to be correlated with HCC [46–48]. The Notch signaling pathway plays a key regulatory role in cellular fate, survival, and injury response in HCC cells [49, 50]. Increasing evidence demonstrates that inhibition of the Notch signaling can enhance the therapeutic efficacy of antitumor agents in HCC cells [51, 52]. As one of the most frequently mutated genes in HCC and other cancers, the tumor suppressor p53 modulates cellular stress conditions and responses to DNA damage and other cytotoxic stresses [53–57]. VEGF, one of the most prominent regulators involved in vasogenesis, was found to be highly expressed in human HCC specimens [58, 59]. Interestingly, we found that the focal adhesion pathway was also enriched in HCC. Activation of the focal adhesion pathway mediated cancer cell survival, invasion, proliferation, and drug resistance [60–62]. And it has been shown to activate different cytoplasmic signaling pathways and co-regulate the pro-survival mechanisms [61, 63, 64]. Integrins are key mediators of cell adhesion molecules which control critical cell functions such as survival and migration by activating certain signaling mechanisms [65, 66]. Given these findings, it is plausible to assume that focal adhesion has a close connection with HCC procession. However, more research is needed to clarify the specific role of the Focal Adhesion Pathway in HCC procession. Indeed, it is essential to further explore the efficacy of the combination of focal adhesion related genes with the long-term survival of HCC patients.

It is an urgent task to seek new biomarkers for early detection, prognostic assessment and decision making in the treatment of HCC. It was found that the abnormal expression of focal adhesion related prognostic genes was associated with poor prognosis of HCC patients [67], suggesting that these abnormally expressed genes may prove to be prognostic biomarkers in HCC. The tumor microenvironment and dysregulation of immune status also impact tumor progression. Increased numbers of studies suggest that the integration of tumor-infiltrating immune cells and clinicopathological characteristics may prove to be a potential prognosis model for predicting the response of patients to immune therapy [68]. In the present study, we found a positive correlation between the risk score and tumor-infiltrating immune cells suggesting that the prognostic genes for predicting the clinical outcomes of HCC patients may be associated with immune cell infiltration. Moreover, our data have also documented that the risk score is associated with the enrichment scores of diverse immune cell subpopulations and related functions, such as aDCs, DCs, iDCs, APC co-stimulation, HLA, and MHC class I, suggesting that the risk score may potentially affect the antigen presentation. Furthermore, we observed that the risk score was relevant to T cell subsets, like Th2 cells and Treg cells, indicating its potential role in regulating the secretion of cytokines from the Th cells.

Knowing that cancer stem cells (CSCs) are highly resistant to conventional chemotherapeutic drugs and radiation therapy, and promote cancer progression owing to their potent self-renewal and invasion capabilities [69, 70], selection of CSCs with specific markers or signaling pathways could be an effective therapeutic strategy for the treatment of chemotherapy-resistant HCC [71]. Of various microenvironmental factors affecting chemotherapy resistance, cell-cell adhesion and communication have recently been identified as key determinants [9, 72]. In the present study, we investigated the expression of prognostic genes with stem-cell-like features measured by RNAss and DNAss. We found that *FYN* and *PPP1CB* were negatively correlated with RNAss, while *PPP1CC* was positively correlated with RNAss, which indicated that prognostic genes might have a relationship with cancer cell sensitivity or resistance to chemotherapy treatment. Moreover, our data showed that increased expression of the prognostic genes was associated with increased drug resistance or sensitivity to a number of chemotherapy drugs. Based on these observations, we preliminarily concluded that overexpression of the prognostic genes could decrease cancer cell sensitivity or resistance to drug treatment and be used as therapeutic targets.

## CONCLUSIONS

In summary, the focal adhesion related gene signature identified in this study showed good performance in different cohorts and could be used to improve the prediction accuracy of OS in HCC patients, though the action mechanism between the focal adhesion related genes and tumor immunity in HCC needs to be further explored. Taken together, would help gain deeper insights into the prognostic value and biological function of focal adhesion related genes in HCC and provide new possibilities for HCC therapeutic intervention.

## MATERIALS AND METHODS

### Data collection

The RNA sequencing expression profile and corresponding clinical information were retrieved from two public cohorts: TCGA-LIHC and ICGC-LIRI-JP. A total of 365 HCC patients with liver cancer datasets were collected from the TCGA portal (https://portal.gdc.cancer.gov/repository). Additional 231 tumor samples were obtained from the ICGC portal (https://dcc.icgc.org/projects/LIRI-JP). The current research follows the access policies and publication guidelines of the TCGA and ICGC cohorts. The 199 focal adhesion related genes were retrieved from the Molecular Signatures Database (MsigDB, https://www.gsea-msigdb.org/gsea/index.jsp) (Supplementary Table 1).

### Establishment of a focal adhesion related gene signature

Using the "limma" R package, DEGs between tumor and adjacent non-tumorous tissues were identified with a false discovery rate of <0.05 in the TCGA cohort. The prognosis genes in HCC patients were screened buy univariate Cox analysis. A protein-protein interaction network of the overlapping prognostic DEGs was constructed with similar interaction patterns grouped. The possibility of interactions between genes was further screened by LASSO algorithm. A prognostic model was constructed with the "glmnet" R package. The basic aim of LASSO was variable selection and some regression coefficients could be strictly equal to 0, thereby obtaining an interpretable model. The response variables were OS and the status of patients in the TCGA cohort, and the independent variables in the regression were the normalized expression matrix of candidate prognostic DEGs. The penalty parameter (λ) for the model following the minimum criteria (i.e. the value of λ corresponding to the lowest partial likelihood deviance) was determined by 10-fold cross-validation, a standard method to estimate the adjustment parameter λ. The risk score of the patients was calculated based on the expression level of each gene and its corresponding regression coefficient by using the following equation: Score = e^sum (each gene’s expression × corresponding coefficient)^. The median risk score was used to classify patients into a high- risk group and a low-risk group. OS of the HCC patients was determined by survival analysis using the "survminer" R package. The predictive power of the model was assessed by time-dependent ROC curve analysis using the "survivalROC" R package. Based on the expression of the prognostic genes, we performed PCA and t-SNE with the "Rtsne" R package to explore the distribution of different groups. Furthermore, univariate and multivariate Cox regression analyses were performed to explore the independent prognostic value of the 5-gene signature.

### Enrichment analysis

To further understand the immune function, ssGSEA was performed to calculate the score of immune cell infiltration and the activity of immune-related pathways between high-risk and low-risk groups using the "GSVA" R package. GO and KEGG analyses in high-risk and low-risk groups were performed using GSEA by GSEA software 4.1.

### Tumor microenvironment and immune response analysis

The infiltration level of immune cell score and stromal cell score was obtained by using the "estimate" R package. The immune score and stromal score associated with the risk score or prognostic model were analyzed by Spearman correlation. Stem-cell-like features with risk score or prognostic model were measured by tumor stemness features extracted from the TCGA tumor samples. Six immune subtypes were defined to measure immune infiltrates. In tumor immune response, differences in prognostic model risk scores between the immune infiltrate subtypes obtained from the TCGA-LIHC were calculated by ANOVA analysis.

### Chemotherapy sensitivity analysis

The NCI-60 database was accessed using the CellMiner interface (https://discover.nci.nih.gov/cellminer/) containing 60 different cancer cell lines from 9 different tumor types. Pearson correlation was used to analyze the association between the prognostic gene expression and drug sensitivity by using 263 drugs approved by the FDA or obtained from clinical trials (Supplementary Table 2).

### Verification of mRNA expression of prognostic genes between HCC and adjacent non-tumorous tissues by qRT-PCR

The qRT-PCR experiments were performed to validate the mRNA expression levels of the five prognostic genes in 20 paired HCC and adjacent non-tumorous tissues recruited from the First Affiliated Hospital of Wenzhou Medical University (Wenzhou, China). This study was approved by the Review of Ethics Committee in Clinical Research of the First Affiliated Hospital of Wenzhou Medical University. Total RNA from the HCC and adjacent normal liver tissue specimens was prepared with the Trizol reagent following the manufacturer’s protocol (Servicebio). Using the RevertAid First Strand cDNA Synthesis Kit (Thermo), RNA was reverse-transcribed into cDNA. Gene expression was normalized to GAPDH. RT-PCR analysis was quantitated with FastStart Universal SYBR Green Master (Roche) by ABI StepOne (Applied Biosystems). The primer sequences were detailed in Supplementary Table 3. Each RNA sampling was performed in triplicate. To compare the expression levels between different samples, the relative expression of focal adhesion related genes was detected by the 2^−ΔΔCt^ method.

### Verification of the protein expression of prognostic genes between HCC and adjacent non-tumorous tissues by IHC

IHC experiments were performed to validate the protein expression levels of the five prognostic genes in 10 paired HCC and adjacent non-tumorous tissues. All specimens were fixed with 10% formalin at room temperature, embedded in paraffin, and consecutively sliced into 4 μm sections. In brief, the tissue slices were firstly deparaffinized, and boiled in 10 mmol/L citrate buffer (pH = 6.4) for 10 min to retrieve the antigen. Then, the sections were treated in methanol containing 3% hydrogen peroxide to inactivate the endogenous peroxidase, and then with citrate buffer (pH = 6.0) to optimize antigen retrieval. The sections were incubated with 1% bovine serum albumin (BSA) in phosphate-buffered saline (PBS) for 30 min to block the unspecific binding. Besides, primary antibodies and HRP-conjugated secondary antibodies were respectively applied to stain the slides. The detail information of antibodies is provided in Supplementary Table 5. After that, the slices were stained diaminobenzidine and counter-stained with hematoxylin. Finally, the sample was sealed, observed, and photographed.

### Statistical analysis

Gene expressions between tumor and adjacent non-tumorous tissues were compared by Wilcoxon test. Differences in proportions were compared by the Chi-squared test. OS in different groups was detected by log-rank test and compared by Kaplan-Meier analysis. Independent predictors of OS were screened by both univariate and multivariate Cox regression analyses. The ssGSEA scores of immune cells or immune pathway activities between high- and low-risk groups were compared by Mann-Whitney test. Spearman or Pearson correlation was used to explore the correlation between prognostic model risk scores or prognostic gene expression levels and stemness score, stromal score, immune score and drug sensitivity. Plots were created using R software (Version 3.6.3) with packages venn, igraph, ggplot2, pheatmap, ggpubr, corrplot, and survminer where appropriate. A two-tailed *p*-value of <0.05 was considered to be statistically significant.

## Supplementary Materials

Supplementary Figures

Supplementary Table 1

Supplementary Table 2

Supplementary Tables 3-5

## References

[1] Zhang L, Ding J, Li HY, Wang ZH, Wu J. Immunotherapy for advanced hepatocellular carcinoma, where are we? Biochim Biophys Acta Rev Cancer. 2020; 1874:188441. 10.1016/j.bbcan.2020.188441 33007432

[2] Chen T, Dai X, Dai J, Ding C, Zhang Z, Lin Z, Hu J, Lu M, Wang Z, Qi Y, Zhang L, Pan R, Zhao Z, et al. AFP promotes HCC progression by suppressing the HuR-mediated Fas/FADD apoptotic pathway. Cell Death Dis. 2020; 11:822. 10.1038/s41419-020-03030-7 33009373 PMC7532541

[3] Llovet JM, Zucman-Rossi J, Pikarsky E, Sangro B, Schwartz M, Sherman M, Gores G. Hepatocellular carcinoma. Nat Rev Dis Primers. 2016; 2:16018. 10.1038/nrdp.2016.18 27158749

[4] Torrecilla S, Sia D, Harrington AN, Zhang Z, Cabellos L, Cornella H, Moeini A, Camprecios G, Leow WQ, Fiel MI, Hao K, Bassaganyas L, Mahajan M, et al. Trunk mutational events present minimal intra- and inter-tumoral heterogeneity in hepatocellular carcinoma. J Hepatol. 2017; 67:1222–31. 10.1016/j.jhep.2017.08.013 28843658

[5] Bruix J, Reig M, Sherman M. Evidence-Based Diagnosis, Staging, and Treatment of Patients With Hepatocellular Carcinoma. Gastroenterology. 2016; 150:835–53. 10.1053/j.gastro.2015.12.041 26795574

[6] Zhang X, Ng HLH, Lu A, Lin C, Zhou L, Lin G, Zhang Y, Yang Z, Zhang H. Drug delivery system targeting advanced hepatocellular carcinoma: Current and future. Nanomedicine. 2016; 12:853–69. 10.1016/j.nano.2015.12.381 26772424

[7] Gao HX, Wang MB, Li SJ, Niu J, Xue J, Li J, Li XX. Identification of Hub Genes and Key Pathways Associated with Peripheral T-cell Lymphoma. Curr Med Sci. 2020; 40:885–99. 10.1007/s11596-020-2250-9 32980897

[8] Lan Q, Wang P, Tian S, Dong W. Mining TCGA database for genes of prognostic value in gastric cancer microenvironment. J Cell Mol Med. 2020; 24:11120–32. 10.1111/jcmm.15595 32818296 PMC7576220

[9] Eke I, Cordes N. Focal adhesion signaling and therapy resistance in cancer. Semin Cancer Biol. 2015; 31:65–75. 10.1016/j.semcancer.2014.07.009 25117005

[10] Nikou S, Arbi M, Dimitrakopoulos FD, Sirinian C, Chadla P, Pappa I, Ntaliarda G, Stathopoulos GT, Papadaki H, Zolota V, Lygerou Z, Kalofonos HP, Bravou V. Integrin-linked kinase (ILK) regulates KRAS, IPP complex and Ras suppressor-1 (RSU1) promoting lung adenocarcinoma progression and poor survival. J Mol Histol. 2020; 51:385–400. 10.1007/s10735-020-09888-3 32592097

[11] Kang HR, Moon JY, Ediriweera MK, Song YW, Cho M, Kasiviswanathan D, Cho SK. Dietary flavonoid myricetin inhibits invasion and migration of radioresistant lung cancer cells (A549-IR) by suppressing MMP-2 and MMP-9 expressions through inhibition of the FAK-ERK signaling pathway. Food Sci Nutr. 2020; 8:2059–67. 10.1002/fsn3.1495 32328272 PMC7174229

[12] Pallasch FB, Schumacher U. Angiotensin Inhibition, TGF-β and EMT in Cancer. Cancers (Basel). 2020; 12:2785. 10.3390/cancers12102785 32998363 PMC7601465

[13] Fousek K, Horn LA, Palena C. Interleukin-8: A chemokine at the intersection of cancer plasticity, angiogenesis, and immune suppression. Pharmacol Ther. 2021; 219:107692. 10.1016/j.pharmthera.2020.107692 32980444 PMC8344087

[14] Landeros N, Santoro PM, Carrasco-Avino G, Corvalan AH. Competing Endogenous RNA Networks in the Epithelial to Mesenchymal Transition in Diffuse-Type of Gastric Cancer. Cancers (Basel). 2020; 12:2741. 10.3390/cancers12102741 32987716 PMC7598708

[15] Hong R, Gu J, Niu G, Hu Z, Zhang X, Song T, Han S, Hong L, Ke C. PRELP has prognostic value and regulates cell proliferation and migration in hepatocellular carcinoma. J Cancer. 2020; 11:6376–89. 10.7150/jca.46309 33033521 PMC7532499

[16] Li J, Hao N, Han J, Zhang M, Li X, Yang N. ZKSCAN3 drives tumor metastasis via integrin β4/FAK/AKT mediated epithelial-mesenchymal transition in hepatocellular carcinoma. Cancer Cell Int. 2020; 20:216. 10.1186/s12935-020-01307-7 32518525 PMC7275473

[17] Atallah J, Khachfe HH, Berro J, Assi HI. The use of heparin and heparin-like molecules in cancer treatment: a review. Cancer Treat Res Commun. 2020; 24:100192. 10.1016/j.ctarc.2020.100192 32673846

[18] Wang L, Gao Y, Zhao X, Guo C, Wang X, Yang Y, Han C, Zhao L, Qin Y, Liu L, Huang C, Wang W. HOXD3 was negatively regulated by YY1 recruiting HDAC1 to suppress progression of hepatocellular carcinoma cells via ITGA2 pathway. Cell Prolif. 2020; 53:e12835. 10.1111/cpr.12835 32557953 PMC7445403

[19] Zheng Y, Long J, Wu L, Zhang H, Li L, Zheng Y, Wang A, Lin J, Yang X, Sang X, Hu K, Pan J, Zhao H. Identification of hub genes involved in the development of hepatocellular carcinoma by transcriptome sequencing. Oncotarget. 2017; 8:60358–67. 10.18632/oncotarget.19483 28947976 PMC5601144

[20] Zhang Y, Wang W, Wang Y, Huang X, Zhang Z, Chen B, Xie W, Li S, Shen S, Peng B. NEK2 promotes hepatocellular carcinoma migration and invasion through modulation of the epithelial-mesenchymal transition. Oncol Rep. 2018; 39:1023–33. 10.3892/or.2018.6224 29399700 PMC5802024

[21] Deng L, Sun J, Chen X, Liu L, Wu D. Nek2 augments sorafenib resistance by regulating the ubiquitination and localization of β-catenin in hepatocellular carcinoma. J Exp Clin Cancer Res. 2019; 38:316. 10.1186/s13046-019-1311-z 31319849 PMC6639974

[22] Chang YY, Yen CJ, Chan SH, Chou YW, Lee YP, Bao CY, Huang CJ, Huang W. NEK2 Promotes Hepatoma Metastasis and Serves as Biomarker for High Recurrence Risk after Hepatic Resection. Ann Hepatol. 2018; 17:843–56. 10.5604/01.3001.0012.3146 30145571

[23] Fu H, He Y, Qi L, Chen L, Luo Y, Chen L, Li Y, Zhang N, Guo H. cPLA2α activates PI3K/AKT and inhibits Smad2/3 during epithelial-mesenchymal transition of hepatocellular carcinoma cells. Cancer Lett. 2017; 403:260–70. 10.1016/j.canlet.2017.06.022 28649002

[24] Guo P, He Y, Chen L, Qi L, Liu D, Chen Z, Xiao M, Chen L, Luo Y, Zhang N, Guo H. Cytosolic phospholipase A2α modulates cell-matrix adhesion *via* the FAK/paxillin pathway in hepatocellular carcinoma. Cancer Biol Med. 2019; 16:377–90. 10.20892/j.issn.2095-3941.2018.0386 31516757 PMC6713643

[25] Yoshihara K, Shahmoradgoli M, Martínez E, Vegesna R, Kim H, Torres-Garcia W, Treviño V, Shen H, Laird PW, Levine DA, Carter SL, Getz G, Stemke-Hale K, et al. Inferring tumour purity and stromal and immune cell admixture from expression data. Nat Commun. 2013; 4:2612. 10.1038/ncomms3612 24113773 PMC3826632

[26] Dib L, San-Jose LM, Ducrest AL, Salamin N, Roulin A. Selection on the Major Color Gene Melanocortin-1-Receptor Shaped the Evolution of the Melanocortin System Genes. Int J Mol Sci. 2017; 18:2618. 10.3390/ijms18122618 29206201 PMC5751221

[27] Hundal J, Miller CA, Griffith M, Griffith OL, Walker J, Kiwala S, Graubert A, McMichael J, Coffman A, Mardis ER. Cancer Immunogenomics: Computational Neoantigen Identification and Vaccine Design. Cold Spring Harb Symp Quant Biol. 2016; 81:105–11. 10.1101/sqb.2016.81.030726 28389595 PMC5702270

[28] Tamborero D, Rubio-Perez C, Muiños F, Sabarinathan R, Piulats JM, Muntasell A, Dienstmann R, Lopez-Bigas N, Gonzalez-Perez A. A Pan-cancer Landscape of Interactions between Solid Tumors and Infiltrating Immune Cell Populations. Clin Cancer Res. 2018; 24:3717–28. 10.1158/1078-0432.CCR-17-3509 29666300

[29] DeSantis CE, Miller KD, Goding Sauer A, Jemal A, Siegel RL. Cancer statistics for African Americans, 2019. CA Cancer J Clin. 2019; 69:211–33. 10.3322/caac.21555 30762872

[30] Meng X, Franklin DA, Dong J, Zhang Y. MDM2-p53 pathway in hepatocellular carcinoma. Cancer Res. 2014; 74:7161–7. 10.1158/0008-5472.CAN-14-1446 25477334 PMC4504428

[31] Iacobazzi D, Garaeva I, Albertario A, Cherif M, Angelini GD, Caputo M, Ghorbel MT. Protein Phosphatase 1 Beta is Modulated by Chronic Hypoxia and Involved in the Angiogenic Endothelial Cell Migration. Cell Physiol Biochem. 2015; 36:384–94. 10.1159/000430257 25967976

[32] Li C, Wu M, Zong G, Wan C, Liu Q, Zhou H, Hua L, Chen Y, Chen X, Lu C. Overexpression of Protein Phosphatase 1γ (PP1γ) Is Associated with Enhanced Cell Proliferation and Poor Prognosis in Hepatocellular Carcinoma. Dig Dis Sci. 2017; 62:133–42. 10.1007/s10620-016-4365-1 27921263

[33] Hervieu A, Kermorgant S. Unconventional role of RAC1 in MET-driven anchorage-independent tumor growth. Mol Cell Oncol. 2020; 7:1803029. 10.1080/23723556.2020.1803029 33235904 PMC7671004

[34] Cui R, Takahashi F, Ohashi R, Yoshioka M, Gu T, Tajima K, Unnoura T, Iwakami S, Hirama M, Ishiwata T, Iwase A, Takahashi K. Osteopontin is involved in the formation of malignant pleural effusion in lung cancer. Lung Cancer. 2009; 63:368–74. 10.1016/j.lungcan.2008.06.020 18752867

[35] Hu Z, Lin D, Yuan J, Xiao T, Zhang H, Sun W, Han N, Ma Y, Di X, Gao M, Ma J, Zhang J, Cheng S, Gao Y. Overexpression of osteopontin is associated with more aggressive phenotypes in human non-small cell lung cancer. Clin Cancer Res. 2005; 11:4646–52. 10.1158/1078-0432.CCR-04-2013 16000556

[36] Romer LH, Birukov KG, Garcia JG. Focal adhesions: paradigm for a signaling nexus. Circ Res. 2006; 98:606–16. 10.1161/01.RES.0000207408.31270.db 16543511

[37] Zhou X, Jiang Y, Li Q, Huang Z, Yang H, Wei C. Aberrant ALOX5 Activation Correlates with HER2 Status and Mediates Breast Cancer Biological Activities through Multiple Mechanisms. Biomed Res Int. 2020; 2020:1703531. 10.1155/2020/1703531 33224971 PMC7673939

[38] Peng Q, Shen Y, Zhao P, Cai S, Feng Z, Cheng M, Wu Y, Zhu Y. Biomarker exploration of microRNA-203 as a promising substrate for predicting poor survival outcome in colorectal cancer. BMC Cancer. 2020; 20:1003. 10.1186/s12885-020-07512-x 33059609 PMC7559172

[39] Pedersen EA, Menon R, Bailey KM, Thomas DG, Van Noord RA, Tran J, Wang H, Qu PP, Hoering A, Fearon ER, Chugh R, Lawlor ER. Activation of Wnt/β-Catenin in Ewing Sarcoma Cells Antagonizes EWS/ETS Function and Promotes Phenotypic Transition to More Metastatic Cell States. Cancer Res. 2016; 76:5040–53. 10.1158/0008-5472.CAN-15-3422 27364557 PMC5010452

[40] Shi W, Ye Z, Zhuang L, Li Y, Shuai W, Zuo Z, Mao X, Liu R, Wu J, Chen S, Huang W. Olfactomedin 1 negatively regulates NF-κB signalling and suppresses the growth and metastasis of colorectal cancer cells. J Pathol. 2016; 240:352–65. 10.1002/path.4784 27555280

[41] Lin X, Li HR, Lin XF, Yu ME, Tu XW, Hua ZD, Lin M, Xu NL, Han LL, Chen YS. Silencing of Livin inhibits tumorigenesis and metastasis via VEGF and MMPs pathway in lung cancer. Int J Oncol. 2015; 47:657–67. 10.3892/ijo.2015.3058 26094984

[42] Reya T, Clevers H. Wnt signalling in stem cells and cancer. Nature. 2005; 434:843–50. 10.1038/nature03319 15829953

[43] Pérez-Plasencia C, López-Urrutia E, García-Castillo V, Trujano-Camacho S, López-Camarillo C, Campos-Parra AD. Interplay Between Autophagy and Wnt/β-Catenin Signaling in Cancer: Therapeutic Potential Through Drug Repositioning. Front Oncol. 2020; 10:1037. 10.3389/fonc.2020.01037 33014767 PMC7461967

[44] Chen C, Nelson LJ, Ávila MA, Cubero FJ. Mitogen-Activated Protein Kinases (MAPKs) and Cholangiocarcinoma: The Missing Link. Cells. 2019; 8:1172. 10.3390/cells8101172 31569444 PMC6829385

[45] Cargnello M, Roux PP. Activation and function of the MAPKs and their substrates, the MAPK-activated protein kinases. Microbiol Mol Biol Rev. 2011; 75:50–83. 10.1128/MMBR.00031-10 21372320 PMC3063353

[46] Cubero FJ, Zhao G, Nevzorova YA, Hatting M, Al Masaoudi M, Verdier J, Peng J, Schaefer FM, Hermanns N, Boekschoten MV, Grouls C, Gassler N, Kiessling F, et al. Haematopoietic cell-derived Jnk1 is crucial for chronic inflammation and carcinogenesis in an experimental model of liver injury. J Hepatol. 2015; 62:140–49. 10.1016/j.jhep.2014.08.029 25173965

[47] Yu L, Wang F, Tai M, Li J, Gong S, Zhou Z, Yin X, Gu X, Li C. 6H2L, a novel synthetic derivative of bifendate, induces apoptosis in hepatoma cells via mitochondrial and MAPK pathway. Eur J Pharmacol. 2020; 882:173299. 10.1016/j.ejphar.2020.173299 32589884

[48] Qian G, Jin X, Zhang L. LncRNA FENDRR Upregulation Promotes Hepatic Carcinoma Cells Apoptosis by Targeting miR-362-5p Via NPR3 and p38-MAPK Pathway. Cancer Biother Radiopharm. 2020; 35:629–39. 10.1089/cbr.2019.3468 32251605

[49] Chatterjee S, Sil PC. Targeting the crosstalks of Wnt pathway with Hedgehog and Notch for cancer therapy. Pharmacol Res. 2019; 142:251–61. 10.1016/j.phrs.2019.02.027 30826456

[50] Butti R, Gunasekaran VP, Kumar TVS, Banerjee P, Kundu GC. Breast cancer stem cells: Biology and therapeutic implications. Int J Biochem Cell Biol. 2019; 107:38–52. 10.1016/j.biocel.2018.12.001 30529656

[51] Chen Z, Zuo X, Pu L, Zhang Y, Han G, Zhang L, Wu Z, You W, Qin J, Dai X, Shen H, Wang X, Wu J. Hypomethylation-mediated activation of cancer/testis antigen KK-LC-1 facilitates hepatocellular carcinoma progression through activating the Notch1/Hes1 signalling. Cell Prolif. 2019; 52:e12581. 10.1111/cpr.12581 30895661 PMC6536599

[52] Fang S, Liu M, Li L, Zhang FF, Li Y, Yan Q, Cui YZ, Zhu YH, Yuan YF, Guan XY. Lymphoid enhancer-binding factor-1 promotes stemness and poor differentiation of hepatocellular carcinoma by directly activating the NOTCH pathway. Oncogene. 2019; 38:4061–74. 10.1038/s41388-019-0704-y 30696957

[53] Jehan S, Zhong C, Li G, Zulqarnain Bakhtiar S, Li D, Sui G. Thymoquinone Selectively Induces Hepatocellular Carcinoma Cell Apoptosis in Synergism With Clinical Therapeutics and Dependence of p53 Status. Front Pharmacol. 2020; 11:555283. 10.3389/fphar.2020.555283 33041795 PMC7522566

[54] Zhou X, Hao Q, Lu H. Mutant p53 in cancer therapy-the barrier or the path. J Mol Cell Biol. 2019; 11:293–305. 10.1093/jmcb/mjy072 30508182 PMC6487791

[55] Tornesello ML, Buonaguro L, Tatangelo F, Botti G, Izzo F, Buonaguro FM. Mutations in TP53, CTNNB1 and PIK3CA genes in hepatocellular carcinoma associated with hepatitis B and hepatitis C virus infections. Genomics. 2013; 102:74–83. 10.1016/j.ygeno.2013.04.001 23583669

[56] Hussain SP, Schwank J, Staib F, Wang XW, Harris CC. TP53 mutations and hepatocellular carcinoma: insights into the etiology and pathogenesis of liver cancer. Oncogene. 2007; 26:2166–76. 10.1038/sj.onc.1210279 17401425

[57] Parrales A, Iwakuma T. Targeting Oncogenic Mutant p53 for Cancer Therapy. Front Oncol. 2015; 5:288. 10.3389/fonc.2015.00288 26732534 PMC4685147

[58] Liu F, Luo L, Wei Y, Wang W, Wen T, Yang J, Xu M, Li B. Association of VEGFA polymorphisms with susceptibility and clinical outcome of hepatocellular carcinoma in a Chinese Han population. Oncotarget. 2017; 8:16488–97. 10.18632/oncotarget.14870 28147320 PMC5369979

[59] Choi SB, Han HJ, Kim WB, Song TJ, Choi SY. VEGF Overexpression Predicts Poor Survival in Hepatocellular Carcinoma. Open Med (Wars). 2017; 12:430–39. 10.1515/med-2017-0061 29318189 PMC5757349

[60] Seguin L, Kato S, Franovic A, Camargo MF, Lesperance J, Elliott KC, Yebra M, Mielgo A, Lowy AM, Husain H, Cascone T, Diao L, Wang J, et al. An integrin β_3_-KRAS-RalB complex drives tumour stemness and resistance to EGFR inhibition. Nat Cell Biol. 2014; 16:457–68. 10.1038/ncb2953 24747441 PMC4105198

[61] Guo W, Giancotti FG. Integrin signalling during tumour progression. Nat Rev Mol Cell Biol. 2004; 5:816–26. 10.1038/nrm1490 15459662

[62] Kim LC, Song L, Haura EB. Src kinases as therapeutic targets for cancer. Nat Rev Clin Oncol. 2009; 6:587–95. 10.1038/nrclinonc.2009.129 19787002

[63] Bosman FT, Stamenkovic I. Functional structure and composition of the extracellular matrix. J Pathol. 2003; 200:423–28. 10.1002/path.1437 12845610

[64] Frantz C, Stewart KM, Weaver VM. The extracellular matrix at a glance. J Cell Sci. 2010; 123:4195–200. 10.1242/jcs.023820 21123617 PMC2995612

[65] Vehlow A, Cordes N. Invasion as target for therapy of glioblastoma multiforme. Biochim Biophys Acta. 2013; 1836:236–44. 10.1016/j.bbcan.2013.07.001 23891970

[66] Brakebusch C, Fässler R. beta 1 integrin function *in vivo*: adhesion, migration and more. Cancer Metastasis Rev. 2005; 24:403–11. 10.1007/s10555-005-5132-5 16258728

[67] Liang X, Xu X, Wang F, Chen X, Li N, Wang C, He J. E-cadherin knockdown increases β-catenin reducing colorectal cancer chemosensitivity only in three-dimensional cultures. Int J Oncol. 2015; 47:1517–27. 10.3892/ijo.2015.3137 26316041

[68] Mauro L, Pellegrino M, Lappano R, Vivacqua A, Giordano F, Palma MG, Andò S, Maggiolini M. E-cadherin mediates the aggregation of breast cancer cells induced by tamoxifen and epidermal growth factor. Breast Cancer Res Treat. 2010; 121:79–89. 10.1007/s10549-009-0456-4 19593637

[69] Huang Z, Cheng L, Guryanova OA, Wu Q, Bao S. Cancer stem cells in glioblastoma--molecular signaling and therapeutic targeting. Protein Cell. 2010; 1:638–55. 10.1007/s13238-010-0078-y 21203936 PMC4875273

[70] Schonberg DL, Lubelski D, Miller TE, Rich JN. Brain tumor stem cells: Molecular characteristics and their impact on therapy. Mol Aspects Med. 2014; 39:82–101. 10.1016/j.mam.2013.06.004 23831316 PMC3866208

[71] Lee HY, Hong IS. Targeting Liver Cancer Stem Cells: An Alternative Therapeutic Approach for Liver Cancer. Cancers (Basel). 2020; 12:2746. 10.3390/cancers12102746 32987767 PMC7598600

[72] Xu X, He Y, Miao X, Wu Y, Han J, Wang Q, Liu J, Zhong F, Ou Y, Wang Y, He S. Cell adhesion induces overexpression of chromodomain helicase/ATPase DNA binding protein 1-like gene (CHD1L) and contributes to cell adhesion-mediated drug resistance (CAM-DR) in multiple myeloma cells. Leuk Res. 2016; 47:54–62. 10.1016/j.leukres.2016.05.007 27258734

