# Supplementary material for: A novel focal adhesion related gene signature for prognostic prediction in hepatocellular carcinoma
Source: Aging (Albany NY). 2021 Apr 13;13(7):10724–48. doi: 10.18632/aging.202871 (PMC8064231; doi:10.18632/aging.202871)
Supplement: Supplementary Figures [file aging-13-202871-s001.pdf]

SUPPLEMENTARY FIGURES

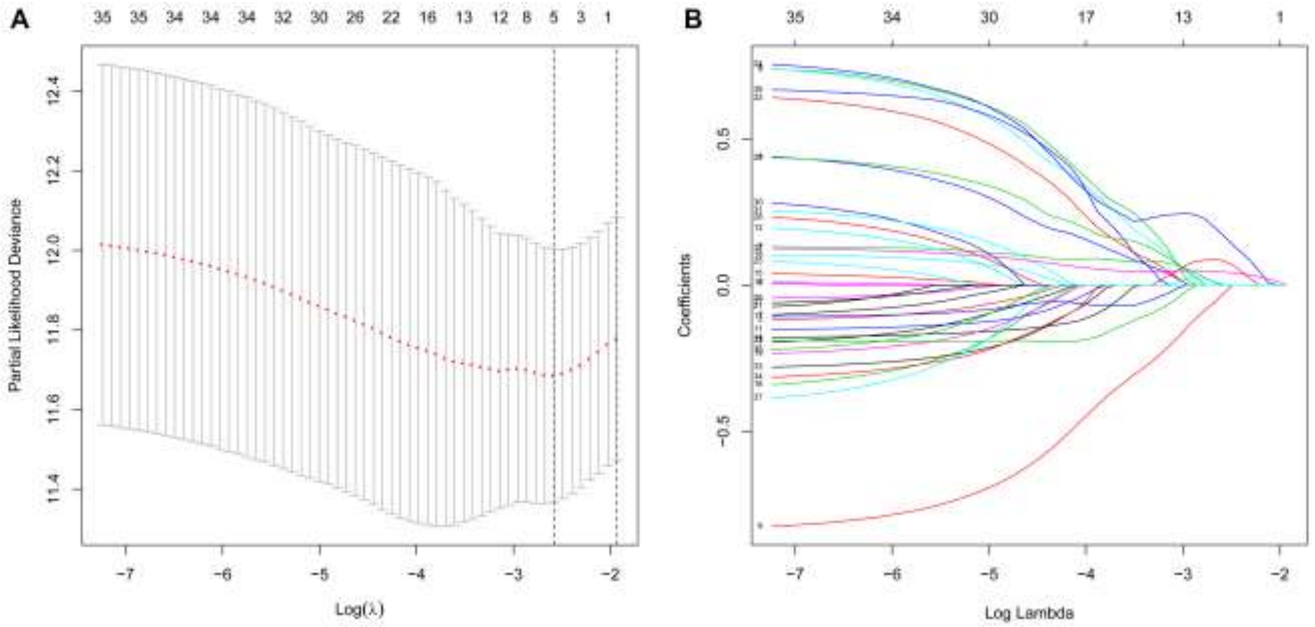

Supplementary Figure 1. Construction of a 5-gene signature model in the TCGA cohort. (**A**) LASSO coefficient profiles of the expression of 37 candidate genes. (**B**) Selection of the penalty parameter (λ) in the LASSO model via 10-fold cross-validation.

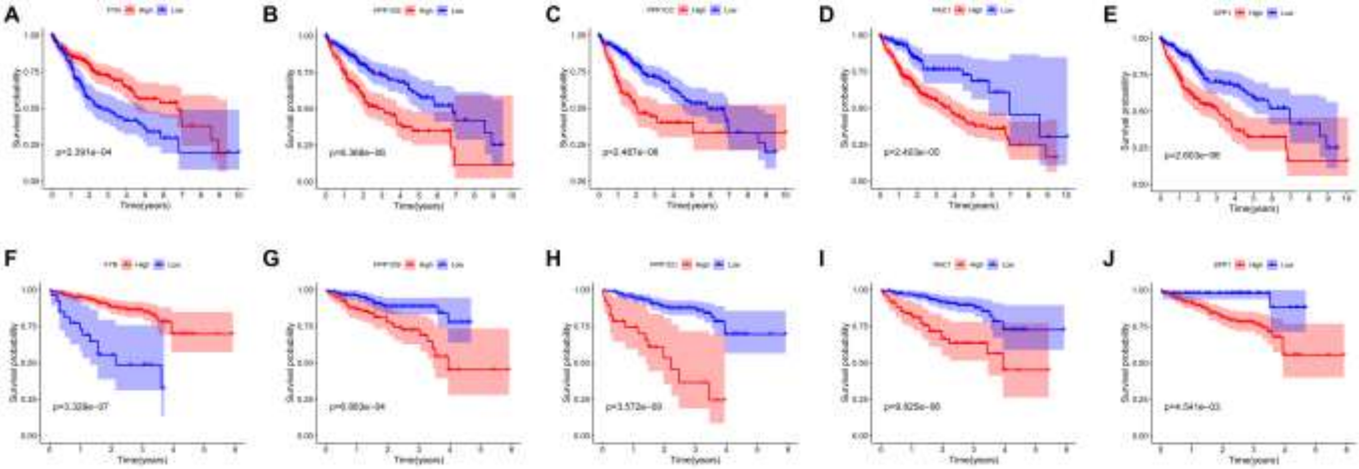

Supplementary Figure 2. Survival analysis of each prognostic gene according to the optimal cut-off expression value. TCGA cohort (**A**–**E**), ICGC cohort (**F**–**J**). All adjusted $P < 0.05$.

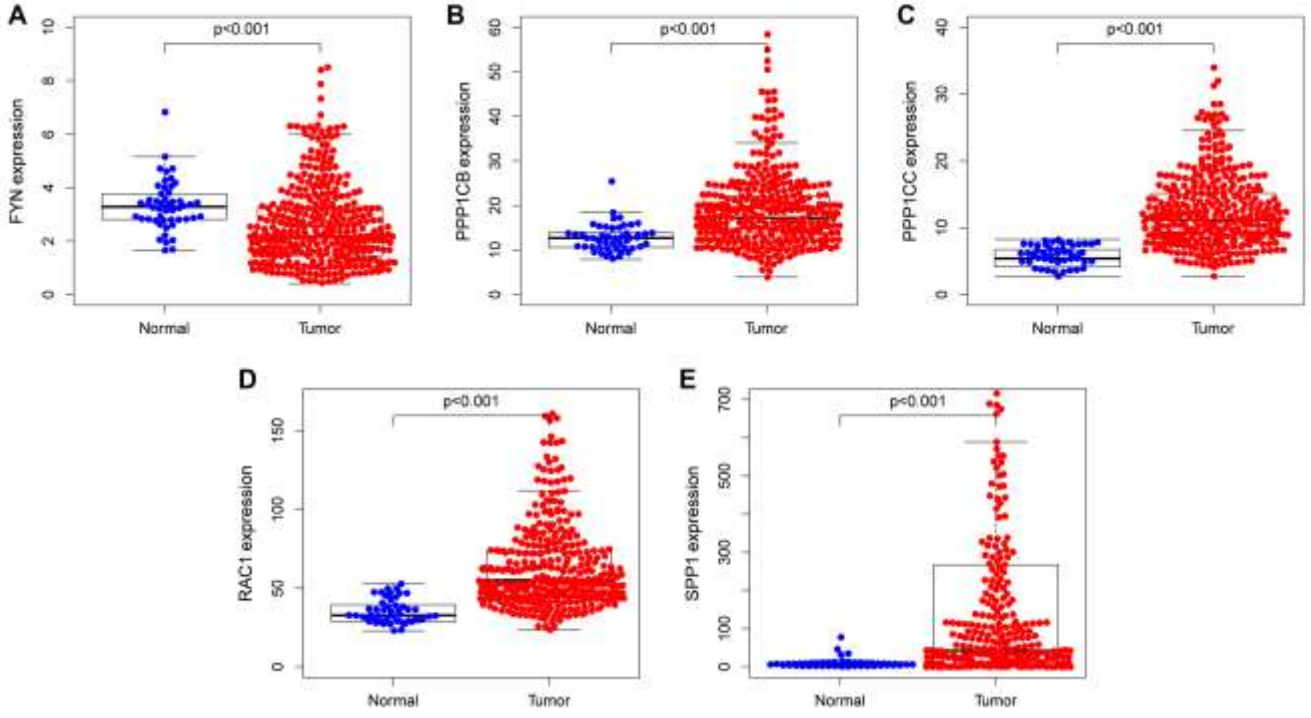

Supplementary Figure 3. Expression of each prognostic gene between HCC and adjacent non-tumorous tissues in TCGA.

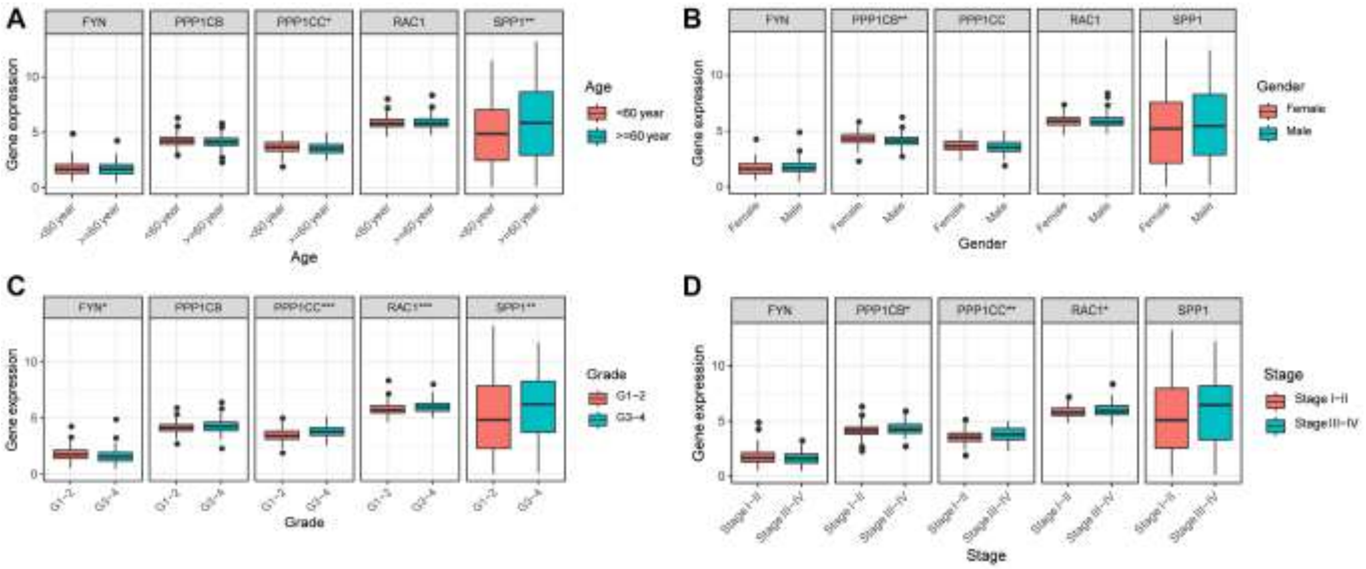

Supplementary Figure 4. Expression level of prognostic genes in different groups stratified by clinical characteristics. (**A**) Age. (**B**) Gender. (**C**) Tumor grade. (**D**) Tumor stage.

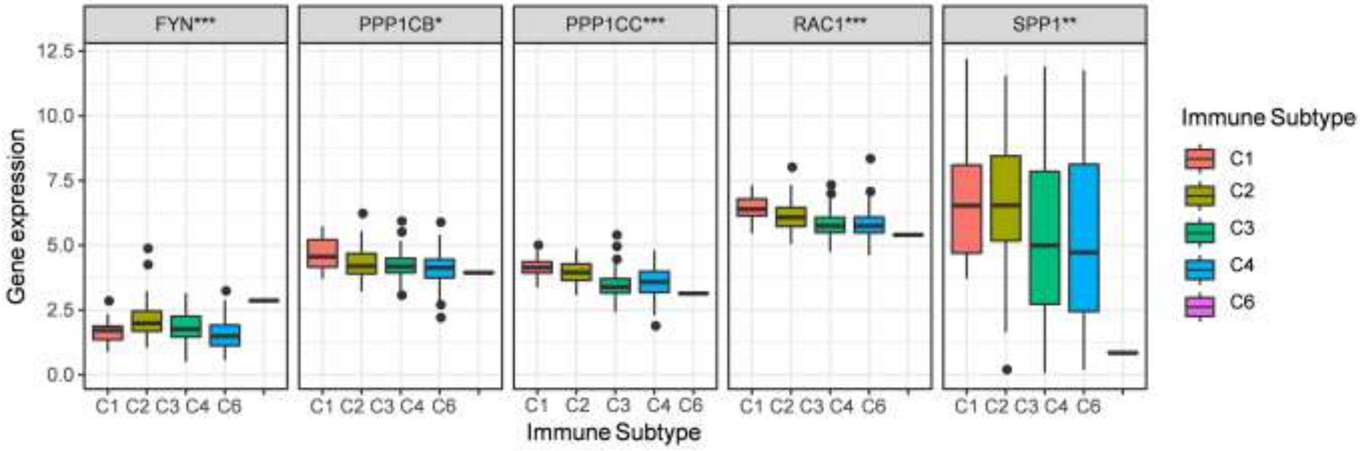

Supplementary Figure 5. Expression level of prognostic genes in different immune infiltrate subtypes.

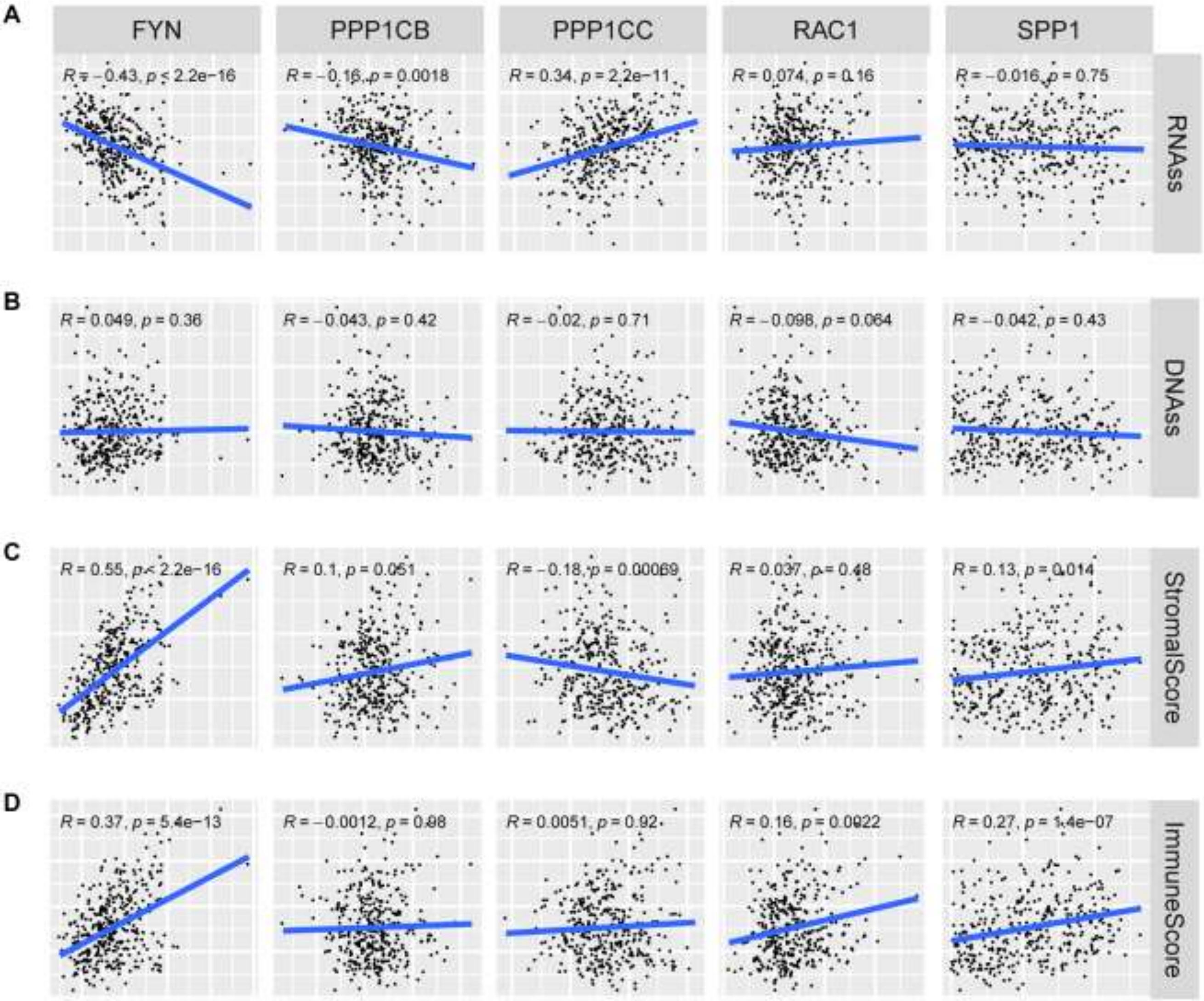

Supplementary Figure 6. Correlation matrixes between the prognostic gene expression and RNAss, DNAss, Stromal Score, and Immune Score.
