# Supplementary material for: A novel focal adhesion related gene signature for prognostic prediction in hepatocellular carcinoma
Source: Aging (Albany NY). 2021 Apr 13;13(7):10724–48. doi: 10.18632/aging.202871 (PMC8064231; doi:10.18632/aging.202871)
Supplement: Supplementary Tables 3-5 [file aging-13-202871-s004.pdf]

SUPPLEMENTARY TABLES

Supplementary Table 3. The primer sequences of the five prognostic genes.

Primers for detection prognostic genes		
**Gene**	**Forward Primer**	**Reverse Primer**
FYN	CCCAACTACAACAACTTCCACG	GCTGGGAATGTAACCTGTCTCTC
PPP1CB	GTCGTCCAGGAAAGATTGTGC	AAGATAGTTGGCTTCTGGTGGG
PPP1CC	AACGGCTGCTGGAAGTGAGA	CACATAGTCCCCAAGAAACAGGTA
RAC1	AGGCCATCAAGTGTGTGGTG	AAGAACACATCTGTTTGCGGA
SPP1	CGAAGTTTTCACTCCAGTTGTCC	AGGTGATGTCCTCGTCTGTAGC
beta-actin	CACCCAGCACAATGAAGATCAAGAT	CCAGTTTTTAAATCCTGAGTCAAGC

Supplementary Table 4. The results of correlation analysis of the prognosis gene expression and chemotherapy drug sensitivity.

Gene	Drug	cor	*P* value
PPP1CC	Chelerythrine	0.441276	0.000417
PPP1CC	AT-13387	0.387333	0.002232
FYN	8-Chloro-adenosine	-0.37588	0.00308
SPP1	Gefitinib	0.375562	0.003107
FYN	PX-316	0.365799	0.00405
PPP1CC	Amonafide	0.36321	0.00434
FYN	Afatinib	-0.35729	0.00507
SPP1	Erlotinib	0.354486	0.005454
SPP1	Lapatinib	0.349174	0.006249
FYN	AFP464	-0.34638	0.006706
FYN	AP-26113	-0.3443	0.007065
FYN	Erlotinib	-0.34236	0.007416
FYN	Nitrogen mustard	-0.34175	0.007529
PPP1CC	Fenretinide	0.340414	0.007782
PPP1CC	Everolimus	-0.33663	0.00854
RAC1	Fluorouracil	-0.33528	0.008826
PPP1CC	Parthenolide	0.334771	0.008936
FYN	Dasatinib	-0.32779	0.010567
FYN	SR16157	-0.3271	0.010741
SPP1	bisacodyl, active ingredient of viraplex	-0.32687	0.010801
SPP1	Vandetanib	0.325235	0.011225
FYN	Bosutinib	-0.32482	0.011335
PPP1CC	Hydroxyurea	0.323699	0.011638
FYN	Ifosfamide	0.321856	0.01215
FYN	AZD-9291	-0.32021	0.012624
FYN	Fluorouracil	-0.31794	0.013304
FYN	Fulvestrant	-0.31674	0.013675
SPP1	Ibrutinib	0.314877	0.01427
PPP1CC	Obatoclax	0.312132	0.015186
SPP1	O-6-Benzylguanine	0.310572	0.01573
PPP1CC	Fludarabine	0.309772	0.016015
PPP1CC	Vorinostat	0.307914	0.016694
SPP1	AZD-9291	0.305134	0.017755
SPP1	Acetalax	-0.30066	0.019585
RAC1	By-Product of CUDC-305	-0.29558	0.021854
FYN	Nelarabine	0.295181	0.022042
FYN	okadaic acid	0.294564	0.022334
PPP1CC	RH1	0.293265	0.022961
FYN	PD-98059	0.293151	0.023017
PPP1CB	Tegafur	-0.29263	0.023272
RAC1	Simvastatin	0.29189	0.02364
FYN	Elesclomol	-0.29039	0.024402
FYN	Chelerythrine	0.289922	0.024641
PPP1CC	8-Chloro-adenosine	0.288427	0.025426
SPP1	Cordycepin	-0.28794	0.025684

FYN	Ibrutinib	-0.28792	0.025698
PPP1CC	Pyrazoloacridine	0.286543	0.026444
RAC1	Denileukin Diftitox Ontak	-0.28369	0.02805
SPP1	Afatinib	0.283317	0.028267
PPP1CC	Palbociclib	0.280656	0.029849
FYN	BML-277	-0.28028	0.030081
SPP1	Dabrafenib	0.278976	0.030886
FYN	Pipamperone	0.278767	0.031018
FYN	Aminoflavone	-0.27842	0.031235
FYN	Salinomycin	-0.27837	0.031268
PPP1CB	Dabrafenib	-0.27821	0.031371
PPP1CB	Entinostat	-0.27667	0.032358
FYN	Vemurafenib	0.276352	0.032566
FYN	Allopurinol	-0.27561	0.033052
SPP1	Tyrothricin	-0.27432	0.033919
PPP1CB	Ixabepilone	-0.27379	0.034277
PPP1CC	Nelarabine	0.273062	0.034778
PPP1CC	Imexon	0.271078	0.036171
FYN	Gefitinib	-0.27104	0.036199
PPP1CC	Cladribine	0.268455	0.038085
PPP1CC	Allopurinol	0.267602	0.038725
PPP1CB	6-Mercaptopurine	-0.26759	0.038735
FYN	Tegafur	-0.26681	0.039326
PPP1CB	Vemurafenib	-0.26558	0.040278
FYN	Sonidegib	-0.26506	0.040684
PPP1CC	Belinostat	0.264338	0.041257
FYN	Everolimus	-0.26137	0.043673
FYN	Vandetanib	-0.2582	0.046388
FYN	1st Precursor Intermediate to TDP 665759	-0.25774	0.04679
SPP1	Cobimetinib (isomer 1)	0.25733	0.047155
FYN	Itraconazole	-0.25568	0.048637
PPP1CC	Cytarabine	0.254939	0.049319
SPP1	Trametinib	0.254883	0.04937

Supplementary Table 5. The detail information of the antibodies used in immunohistochemistry.

Protein	Antibody Name	Antibody Number	Company
FYN	Anti-FYN antibody [D2-A10]	EM1709-88	HUABIO
PPP1CB	Anti-PPP1CB antibody [A7-C7]	EM1706-97	HUABIO
PPP1CC	PPP1CC Polyclonal Antibody	11082-1-AP	Proteintech
RAC1	RAC1 Monoclonal Antibody	66122-1-Ig	Proteintech
SPP1	Osteopontin Polyclonal Antibody	22952-1-AP	Proteintech
